# The Week's Work

**Published:** 1910-05-07

**Authors:** 


### The Week's Work.

### HOSPITALS AND THE PRESS.

The relation between the Press and charitable institutions is a matter which needs the closest consideration of everyone interested in either journalism or charitable work. From the hospital point of view it is a subject that has been frequently discussed in these columns as well as in papers read before associations of hospital and institutional workers; but, so far as we know, there has not been a hearty discussion in which journalists as well as hospital men have participated. To some extent the claims of the two sections appear to clash, but we venture to think that this disagreement is merely apparent and not by any means real. The journalist has no ill-feeling against hospitals; he has no ill-feeling against the medical profession or against nurses or against hospital workers. His object is merely to cater for the tastes of his readers, and it is just as well that medical men and institutional workers should recognise this fact. If the institutional worker took care to keep the Press thoroughly well informed of what was going on in the hospital world, the newspapers might be made into great allies instead of periodically seeming to attack the hospital and the system of charitable relief it represents. At present it is left to the newspaper to take the initiative. If there is an accident the reporter appears at the hospital gates, where he is as out of place as he would be at a German Surgical Congress. The paper makes the mistake, more than once, of sending a reporter whose acquaintance with hospitals is of the vaguest, a man who does not know where to turn for information, who takes the superintendent for the porter and the porter for the secretary, the house surgeon for a student, and the matron for the ward maid. The result of his visit in search for information is often hugely unsatisfactory. Unsatisfactory from the paper's point of view, because he has found out less than the public wants to know, and unsatisfactory from the hospital's, because he has worried everyone into an ill-temper.

### THE PRESS AND SCANDALS.

The evil results of this want of system and cooperation are, fortunately, not so serious as they might be, but they are well seen on those occasions when there is a newspaper storm about some allegation of ill-treatment at one of our large hospitals. And here it may be allowable to express a wish that in the future newspapers would take up the cudgels for the hospital with as much vigour as hitherto they have entered the lists on behalf of Boards of Guardians and individual patients who have felt themselves aggrieved by their treatment in hospital. When a hospital scandal supplies a daily with effective scare headlines, a special reporter is told off to obtain the particular hospital's opinion on the case; very often he is told to make the round of half a dozen institutions and get the opinions of their secretaries on the scandal, and these are printed in all haste, frequently without giving the men who have expressed the opinions a chance of seeing in what shape their statements have been taken down by the zealous journalist who interviewed them. The next day appears the counter statements and opposite opinions of the Guardians, the victims, or the scandalmongers, as the case may be, and so the controversy drags on for nine days or more, until the public finds something new in which to interest itself. All the publicity does no one any good; but it is necessary from the paper's point of view, and, being necessary, has to be secured at all costs. Again, a newspaper gets hold of some part of the truth in the matter of a novel method of treatment. A reporter is at once sent to investigate, and he ferrets out whatever he can obtain. Often the information he brings back and works into his copy is of the most ludicrous character. Thus, who has not smiled at the peculiar paragraphs concerning vaccine and serum treatment which have appeared from time to time in the papers, at the wonderful reports of operations, and, still more lately, at the exceedingly misleading descriptive studies on the use of spinal anæsthesia at one of our London hospitals? It may be said that this is a matter which journalists should take up themselves, and that no reporter worth his salt will undertake to write "a story" on something unless and until he has attempted to read up the subject and made himself more or less conversant with its ins and outs. That certainly holds in the case of special articles which appear in the better-class papers, but it equally certainly does not hold with regard to the small paragraphs of the nature just stated. In the circumstances it is reasonable to expect that hospitals should do something to safeguard their own interests, and the best way of securing this is to co-operate with the papers in a systematic and dignified manner.

### A CENTRAL HOSPITAL PRESS BUREAU.

In America the relations between the Press and the hospitals are by no means so amicable as they are here, and hospital superintendents express themselves very strongly when their opinion is asked about the advisability of giving information to the papers. To a large extent this enmity, which is regrettable, no matter from what point one looks at the matter, is due to the well-known hustling methods of the American pressman, who has long since given up the idea that there exists any privacy for people who are "in the public eye." The American pressman will go to lengths far exceeding those of his English, French, or German *confrère* to obtain hospital news. He will get affidavits from the porters, and when a hospital scandal is in question a discharged employé of the institution is a God-send to him. Is it to be wondered at that the superintendent loses his temper and his patience alike and refuses point blank to have anything to do with the Press? A recent New York scandal, in which the food of the patients was subjected to keen and, let us add, very ignorant criticism, led to the issue of an order by the authorities at one hospital that no worker was to be allowed to talk to a pressman. Notwithstanding these strict injunctions a reporter managed to get copy out of one or two of the staff, and these members, it is interesting to note, have since been suspended pending an investigation of their case by the House Committee. To institutional workers there is nothing drastic in this suspension. It is highly improper that members of the staff or employés should discuss debatable matters with representatives of the Press. All inquiries should be made at the secretary's office, and the secretary or the superintendent is the proper individual to supply all information. There can be no two opinions as to the advisability of making one individual responsible for what is given out to the Press and of limiting the "interview area" in which the speculative cross-questioning of the reporter may be allowed full vent to a zone entirely inside the secretary's office. The secretary's office ought to be the Press Bureau of the institution, and here, and here only, newspaper representatives ought to be seen. Any interviews they may wish to have with members of the staff or employés, if sanctioned by the secretary, should be held here, and the finished paragraph or news report should be written out here and initialled by the secretary. If that were done we would see much less of the absurd reports and news items in which hospitals figure so largely in the daily newspapers. In the future, too, it may be necessary to consider the advisability of establishing a central hospital Press Bureau for every large city, such as London or Manchester, where there are many hospitals with more or less common interests. Such a bureau would have charge of all the publicity arrangements of the institutions. It would be in charge of a man with journalistic and secretarial experience, who, when any information is wanted, can lay his hand on exactly the points most desired. Such a bureau, well managed and well supported, ought to be of immense help and service to the voluntary hospitals in this country.

### A BELGIAN PIONEER.

The first number of *L'Infirmière*, a new Belgian nursing paper, publishes, appropriately enough, a biographical note, with portrait, of the late Dr. Cæsar Paepe, who may be described as the pioneer of nursing reform in Belgium. Paepe was born in 1842, and was well known as a specialist in social hygiene and prophylactic medicine. He gave much attention to the nursing problem in France, Germany, and England, and was a well-known writer on institutional subjects. In 1887 he started a course of lectures for hospital nurses in Belgium, and his classes soon became popular. They were well attended and directed public attention to the urgent need for reform in the Belgian nursing service. The result of Dr. Paepe's efforts have been eminently successful, and the creation of *L'Infirmière itself is pro*of that the interest he tried to encourage and stimulate is not waning, but has gathered strength and increased to the benefit of the institutions no less than to that of the nursing profession. In the same number, Miss Cavell, *directrice* of the Belgian Nursing School, writes an article on "The Duties of the Nurse," which is concise and clear. A third paper is from the pen of Dr. Geraerd on the care of contagious cases.

### HOSPITALS AND THE LAW OF NEGLIGENCE.

At the last meeting of the Medico-Legal Society Mr. Ross Brown read an interesting and elucidative paper on the liability of hospitals and hospital authorities in actions for negligence. The subject is so important that we hope to give a full abstract of the paper and the cases dealt with in next week's issue of The Hospital. At present the position of the hospital with reference to the general public is one that stands in need of some comment and elucidation. The public has become familiar enough with actions for malpraxis and damages for negligence in private practice, and every medical man has to reckon with this familiarity when he undertakes the treatment of a case. The result has been, in the main, beneficial both to the profession and to the public. To the former, in so far as in doubtful or complicated cases no practitioner is likely to leave untried the best modern means of diagnosis, such as, for instance, an examination under the $x$-rays in every case where fracture or dislocation is a possible complication to an external injury. To the public, in so far that it has learned that these new and up-to-date methods demand additional expense and consequently more costly treatment. In the case of institutions, however, the position is not so clear. There are a few cases which may be cited which go far to support the theory that no action for negligence can lie against a charitable institution, but they do not by any means exhaust the possibilities of the subject, and there are conceivable conditions in which the verdict may not be so entirely favourable to a hospital. In any case hospital authorities ought to be well conversant with reported cases and with the law as it stands, and for that reason such papers as that contributed by Mr. Ross Brown are to be welcomed as expert expositions of the subject which are useful for reference purposes. Such papers draw the attention not only of the profession and of hospital men, but of the public as well to certain points on which it is impossible to harp too much.

### THE DRUG MARKET.

On the whole business in the drug market has been quiet, but a large business has been done in quinine, as a result of the higher prices paid for cinchona bark at the recent sales in Amsterdam, the feeling being that makers might advance their prices for quinine by ½d. per oz.; up to the present, however, no advance in price has taken place. Rhubarb is rather low in price at present, and this appears to be a good time to buy. Castor oil is again dearer. Both citric acid and cream of tartar are tending rather dearer. There has been no reduction in the price of calomel or other salts of mercury. The prospects of the new crop of belladonna leaves are good, but prospects as regards hyoscyamus are unfavourable. The price of morphine is maintained at present, but it is not altogether improbable that a reduction may be made before long.

